# Two-Dimensional One-Atom-Thick Gold Grown on Defect-Engineered Graphene

**DOI:** 10.1021/acsnano.5c01538

**Published:** 2025-06-10

**Authors:** Wael Joudi, Sadegh Ghaderzadeh, Alberto Trentino, Kenichiro Mizohata, Kimmo Mustonen, Elena Besley, Jani Kotakoski, E. Harriet Åhlgren

**Affiliations:** † Department of Physics, 3835 University of Helsinki, Pietari Kalmin katu 2, 00560 Helsinki, Finland; ‡ Faculty of Physics, 27258 University of Vienna, Boltzmanngasse 5, 1090 Vienna, Austria; § Vienna Doctoral School in Physics, University of Vienna, Boltzmanngasse 5, 1090 Vienna, Austria; ∥ School of Chemistry, 6123 University of Nottingham, NG7 2RD Nottingham, U.K.

**Keywords:** 2D metal, metallene, 2D gold, goldene, graphene, transmission electron microscopy, ultralow-energy ion irradiation

## Abstract

In this work, a general route to creating two-dimensional, one-atom-thick metal layers, metallene, on functionalized graphene is proposed. To explore its viability, low-energy ion irradiation is performed to introduce vacancies into initially pristine graphene, followed by ultralow-energy gold irradiation to deposit individual gold atoms onto it. While gold freely migrates on pristine graphene, vacancies provide anchoring points where gold atoms gather and promote the growth of atomically thin nanoplatelets. The physical and chemical structure of the gold flakes is confirmed through atomic-resolution scanning transmission electron microscopy and electron energy loss spectroscopy, while their formation is investigated using ab initio simulations. The thickness and diameter of the gold flakes are directly proportional to gold ion fluence during ultralow-energy ion irradiation. Gold atoms in small gold structures are arranged in a one-atom-thick hexagonal lattice. Larger goldene platelets with lateral sizes in the range of tens of nanometers contain multiple gold layers. Mono- and few-layer flakes are metastable under continuous 60 keV electron irradiation during imaging but occasionally rotate and take small jumps as the atoms at the edges move. A reversible transformation between a flat monolayer and an amorphous three-dimensional gold cluster is observed in the experiments and is also seen in the simulations.

## Graphical abstract

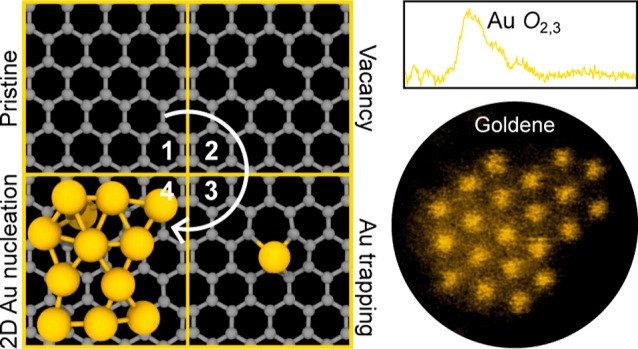

## Introduction

Metallenes—atomically thin structures composed only of metals—are a recent addition to the family of two-dimensional (2D) materials. Although metallenes are by definition one-atom-thick versions of their bulk counterparts,^1^ in practice, the term is often used to describe structures that are nanometers thick. This is because the synthesis of truly atomically thin metals has turned out to be a formidable challenge. Indeed, most metal structures arise from metallic bonding based on the free-electron behavior of their highest occupied *s* valence states, which favors three-dimensional close-packed crystal structures. As a result, increasing the surface area by requiring a 2D arrangement of the atoms leads to increasing surface energy and thermodynamic instability.^2^

Nevertheless, metallenes remain an active research topic due to their expected superior properties ranging from bendability to increased catalytic performance^3–6^ to new electronic and optical properties arising from the confined electronic structure.^7^ They may also find uses in applications where metal nanoparticles are already employed, such as in biomedicine, thermoplasmonics,^8^ and optoelectronics.^9^

Due to the advanced plasmonic and catalytic properties of gold nanostructures,^10^ goldene (atomically thin gold) provides an especially attractive target for metallene synthesis. Additionally, it was discovered more than two decades ago,^11^ that gold atoms have an alternative way to form bonds through s-p-d orbital hybridization^12^ that allows the formation of free-standing flat gold structures. However, for structures of 12 atoms or more, the planar arrangement becomes disfavored over a three-dimensional shape.^13^ Nevertheless, the synthesis of goldene has been tried with a variety of methods, including spatial confinement,^14^ chemical etching,^15^ mechanical thinning,^16^ electron-beam-induced thinning,^17^ and self-assembly.^18^ So far, one of the closest structures to goldene is the two-atom-thick metallene alloy of copper and gold that was created on hexagonal boron nitride and graphene by evaporating gold onto a material already containing copper.^19^ However, this structure consists of a monolayer of gold and a monolayer of copper atoms on top of each other, and it is very sensitive to the electron beam. Pure gold structures, instead, grow into three-dimensional shapes on van der Waals materials.^20^ Interestingly, a way to promote the planar arrangement of gold was suggested in a theoretical study in 2007 by Walter et al.^21^ The authors showed that a defective surface with trapped electrons can enhance the bonding of metal atoms and stabilize flat gold structures of at least up to 20 atoms. This may also explain the observation of free-standing flat gold nanoribbons at a graphene edge^22^ and thin gold structures on graphene forming under continuous electron irradiation,^23^ possibly assisted by tensile strain that has also been shown to stabilize metallene structures.^24^

Here, this study systematically follows this approach by first functionalizing free-standing graphene using low-energy ion irradiation (^197^Au^+^ at 200 eV) to create a substrate with vacancies providing dangling bonds to both trap gold atoms and to promote the 2D growth of goldene. These gold atoms, however, pass through the sample and are lost in the vacuum during the process. Then, additional gold ions are landed gently onto the surface using ultralow-energy (25 eV) ion irradiation. These ions are expected to become immediately neutralized on graphene that is an extremely good conductor, and then migrate as neutral atoms on the clean surface until they encounter a vacancy or a growing gold structure where they become attached. This results in the growth of one-atom-thick gold with lateral sizes on the order of a few nm. When the amount of gold is increased, the structures grow both in thickness as well as in lateral size. The physical and chemical characteristics of the gold structures are unambiguously revealed by atomic-resolution scanning transmission electron microscopy (STEM) and electron energy loss spectroscopy (EELS). Both the formation and the defect-mediated stability of the 2D gold structures are explained by ab initio-based atomistic simulations.

## Results and Discussion

### 2D Gold

The free-standing graphene samples were prepared by transferring commercial samples grown via chemical vapor deposition (CVD) onto microscopy grids made either of Si with a perforated SiN window on top or of Au with a holey amorphous carbon film. After the transfer, the samples were exposed to ^197^Au^+^ irradiation in ultra high vacuum with an ion implanter at an energy of 200 eV and a fluence of ca. 1 × 10^14^ cm^–2^. These conditions are known to create vacancies in graphene,^25^ leading to its functionalization. Previous studies have shown that such functionalized graphene can be used to host individual impurity atoms when followed by ultralow-energy (25 eV) irradiation with Au^25^ or physical vapor deposition of Al, Ti, Fe, Ag, or Au.^26^ Next, the additional gold was introduced by ultralow-energy irradiation^25^ at the same fluence as the initial ion irradiation step. While in the previous work, the samples were exposed to laser irradiation after the second irradiation step to maximize the area of clean graphene and increase the number of visible single-atom impurities, this step is now skipped to allow a direct view of the as-grown structures inspired by the predicted promotion of flat growth on defective substrates.^21^ The experiments were repeated at different fluences of up to 1 × 10^16^ cm^–2^. As shown in the annular dark field (ADF) STEM image in Figure 1a, this leads to the growth of nanometer-sized gold structures with varying shapes, including atomically thin gold flakes, which were not seen in the previous studies.

**Figure 1:**
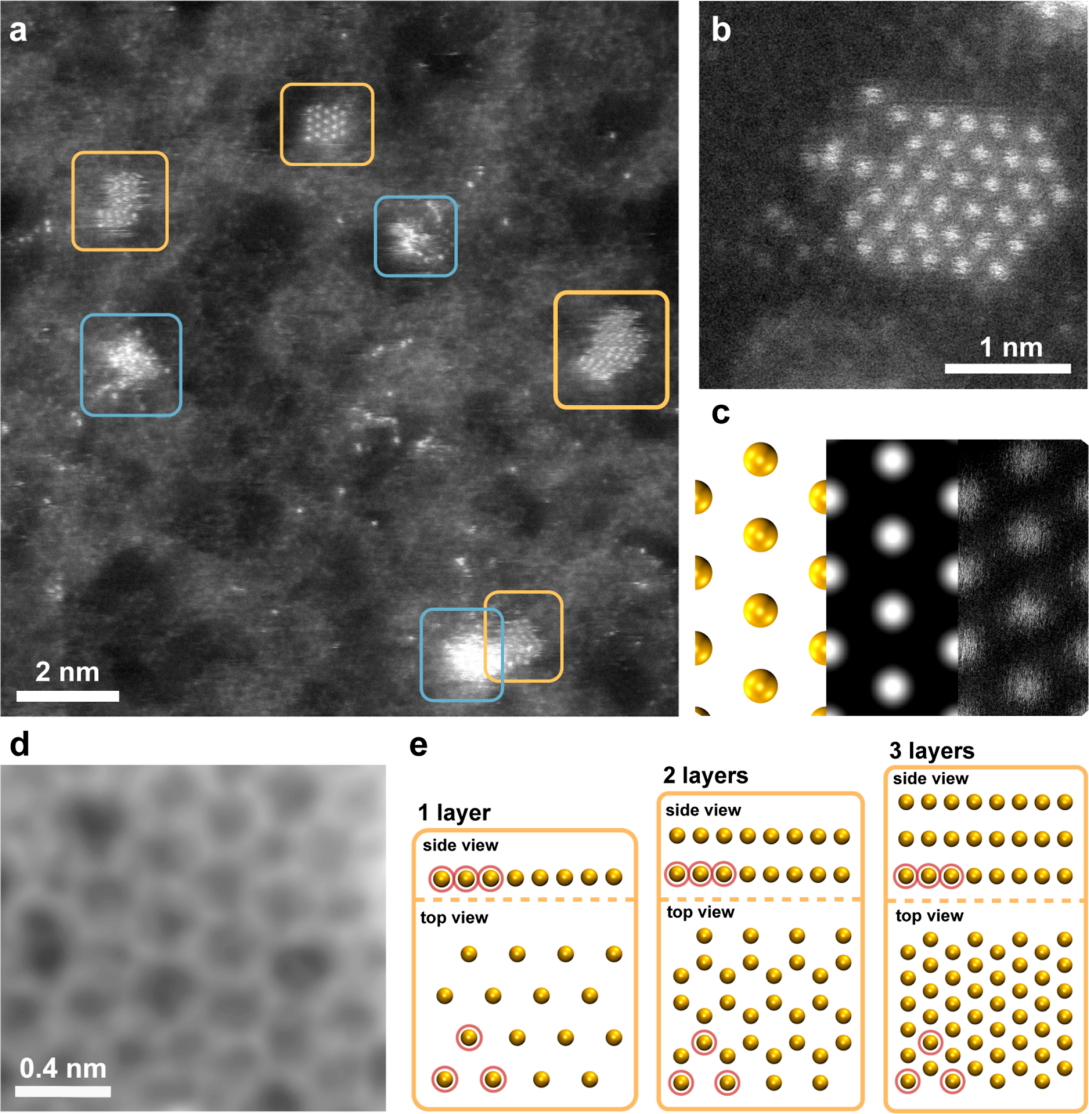
One-atom-thick 2D gold flakes on graphene. (a) Medium-angle annular dark field (MAADF) STEM overview of the sample with multiple 2D gold clusters encircled with yellow, some of which are moving under the beam during imaging and appear blurred. The sample also shows individual Au dopants and three amorphous clusters, the latter of which are encircled in blue. The gray areas contain carbon-based contamination on either side of the graphene membrane, and the black areas contain atomically clean graphene. (b) MAADF STEM image of a 2D gold flake. Gold atoms appear bright, with stark contrast to almost black graphene. (c) Left to right: single-layer Au FCC(111) model, the corresponding simulated STEM image, and the raw MAADF STEM image recorded at 60 kV. (d) MAADF STEM image of the defect-engineered graphene with vacancy defects created with an ion irradiation dose of 1 × 10^16^ cm^–2^. The image is filtered with Gaussian blur of 8 pixel radius. (e) Lattice model of atomically thin gold showing 4 × 4 unit cells with a thickness of one, two, and three layers in FCC(111) orientation with top and side views. Three atoms in the bottom layer are highlighted with red circles in each image for comparison.

### 2D Monolayer Gold

An example image of a 2D single-atom-thick gold flake is shown in Figure 1b. This structure contains 36 gold atoms in a single layer arranged into a hexagonal lattice. The image contains three different intensity regimes. The brightest features correspond to the gold atoms of the cluster. A side-by-side comparison of the one-layer model, the corresponding STEM image simulation, and an experimental STEM image is shown in Figure 1c. The darkest areas at the top and left edges correspond to atomically clean graphene, where very faint 6-fold symmetry in the spatial intensity distribution can be observed. The large difference in brightness is a result of the local charge difference of the atomic nuclei, where the heavier gold scatters more electrons than the lighter carbon.^27^ The intermediate intensity between graphene and gold originates from amorphous carbon-based surface contamination. The larger scattered intensity of the carbon-based contamination compared to that of the clean single-layer graphene is due to the differing thickness of the two carbon-containing structures. In the overview image (Figure 1a), four similar one-atom-thick 2D gold flakes are encircled with orange frames. Some of these are not stable when the electron beam scans over the sample, which leads to the horizontal scan artifacts in these areas. The areas highlighted with blue frames contain amorphous quasi-spherical gold nanoclusters. An example image of the functionalized graphene is shown in Figure 1d. This image was recorded on a sample irradiated with low-energy ions (at 200 eV) at a fluence of 10^16^ cm^–2^. The lattice contains multiple vacancy-type point defects in the freestanding monolayer, as expected.

The largest one-atom-thick gold flakes consist of approximately 40 gold atoms and are a few nanometers in size. The monolayer structure is clearly distinct from thicker atomic arrangements, as seen in Figure 1e. It has a hexagonal pattern with an atom in the middle, and a longer interatomic distance in the top-down projection than in the multilayer structures as the sublattices seen for 2- and 3-layer cases are missing. In the double layer, goldene has a honeycomb structure, while the triple layer and thicker arrangements display the conventional FCC(111) projection where the middle of the hexagon is also filled. Comparison of the experimental images of the single-layer gold structure to simulated images based on the atomic model of single-layer gold produces a clear match, as seen in Figure 1c. On the other hand, the experimental images in Figure 3b,c show a structure with two layers and at least three layers, respectively, and match with the image simulations presented in Figure 3d for the respective layer numbers. The average distance between gold atoms in the monolayer structure was measured at 0.28 ± 0.02 nm, see Figure 2a–b. The uncertainty arises from image-to-image variations due to the metastable nature of the flake under the 60 kV electron beam, as will be discussed below. These values are in line with previously reported lattice parameters for thin gold structures (0.24 nm,^16,18^ 0.26 nm,^28^ 0.262 nm,^15^ and 0.28 nm^23^).

**Figure 2:**
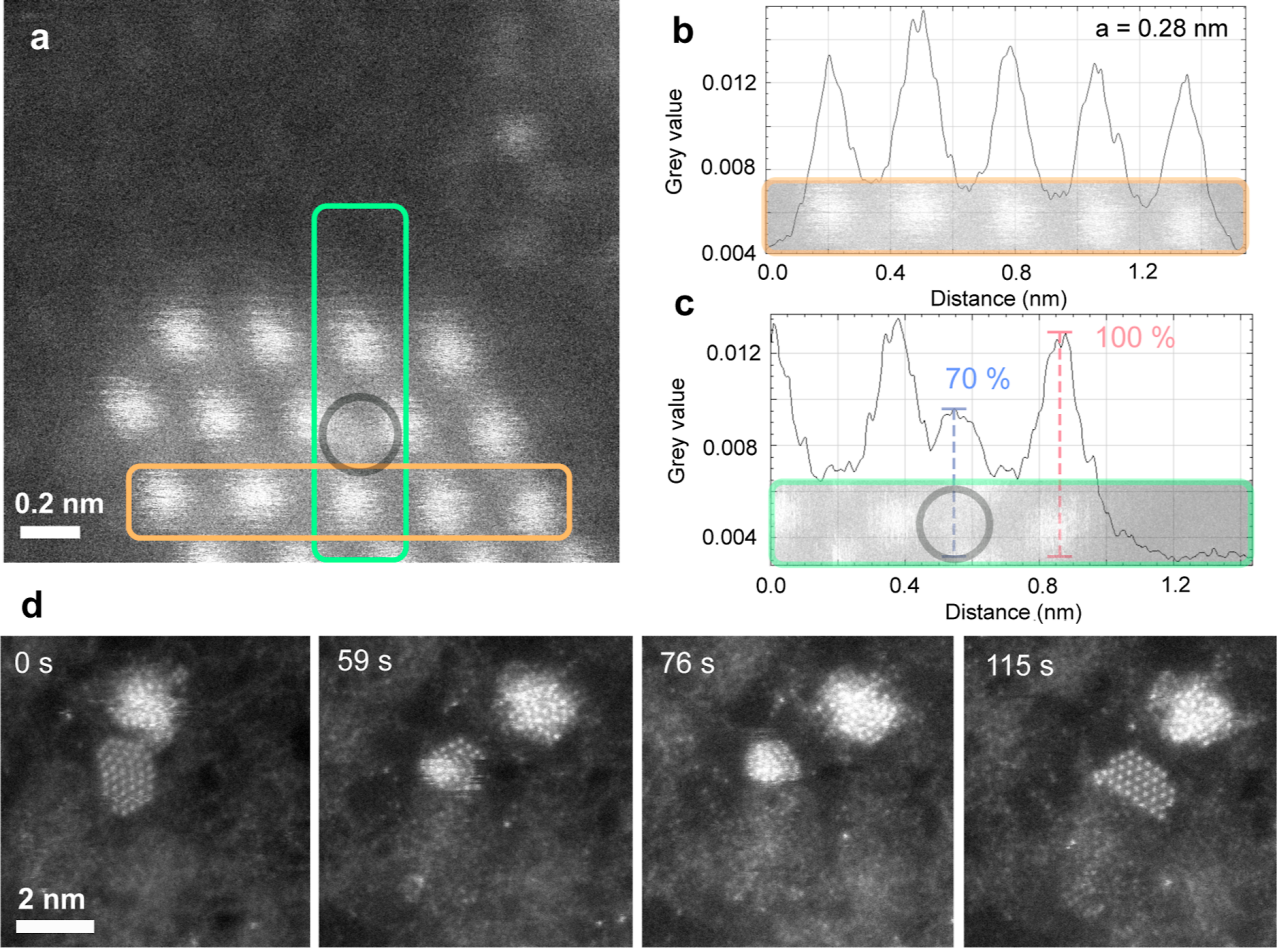
Lattice parameter of 2D Au, adatom, and dynamic behavior. (a) MAADF STEM image of 2D gold on graphene. (b) Intensity profile along the orange box in panel (a) gives an average lattice parameter of 0.28 nm. (c) Mobile adatoms appearing on the gold surface. The intensity profile along the green box of panel (a) shows the adatom intensity to be 70% of the intensity observed for the gold atoms. (d) Electron beam-driven transformation from 2D Au to 3D and back to 2D. Snapshots of the raw MAADF STEM time series show the collapse of a fully 2D gold monolayer into a spherical cluster and back into the monolayer configuration under a 60 kV electron beam. A second, larger cluster in the top right corner remains amorphous throughout the imaging.

It is important to note that the formation of flat one-atom-thick gold was not observed when gold was introduced onto pristine graphene. In this case, the gold forms spherical nanoclusters, which are also occasionally observed in this study alongside the flat gold flakes. On the pristine surface, a compounding species such as Cu is needed to form a 2D layer, resulting in 2D CuAu.^19^ Indeed, an Au adatom placed on top of pristine graphene is mobile at room temperature with the binding energy of 0.51 eV.^26^ To form a stable configuration, the gold atom requires a trapping site, such as a vacancy or hydrocarbon contamination^29^ that naturally occurs on the surface of graphene in ambient conditions.^30–32^ However, of these two possibilities, only the vacancy promotes the growth of flat 2D goldene due to its atomically flat nature.

Interestingly, our 2D gold structures sometimes contain individual impurity atoms. One such case is highlighted by a gray circle in Figure 2a. The impurities are mobile during continuous electron irradiation, which makes spectroscopic confirmation of their element practically impossible. A comparison between the intensity line profiles of Figure 2c highlights the presence of an impurity atom, which has approximately 70% of the intensity of gold. Based on the intensity difference being similar to what has been measured in 2D CuAu structures,^19^ it is likely this impurity atom is copper. Finding copper on graphene grown by CVD is not uncommon, as residual copper may remain from the growth process on the copper catalyst.

The intensity of gold atoms in the MAADF STEM image varies by ca. 7% of the recorded mean Au intensity in the image. This cannot explain the 30% reduction in the intensity observed for the impurity atom. Another possibility that could, in principle, lead to misinterpretation arises from the defocus of the electron beam when atoms are at different depths (i.e., an atom is out of focus). However, the embedded anchoring atom is only 1.11 Å lower compared to the rest of the gold structure according to the ab initio calculations (discussed below). Even if the atom were to be on the opposite side of the graphene membrane, the difference in the depth would be only on the order of 0.5 nm. Such differences are too small to cause an observable intensity change under our imaging conditions.

Next, the stability of the 2D gold under our imaging conditions (room temperature, 60 keV electron beam, and 10^–10^ mbar pressure) was tested. During imaging, the monolayer clusters occasionally rotate, and atoms at the edges take small jumps. As a result, the flakes can move locally in the range of several Å, demonstrating a weak bonding to graphene visualized in the video V1 of the Supporting Information. Movements of small gold clusters on graphene on Ru(0001) have been reported in the literature.^33^ However, the 2D gold flakes observed here do not move distances larger than the size of the flake, in contrast, to, for example, noble gas clusters within double-layer graphene.^34^ This indicates that they remain attached to the defect site. Occasionally individual gold atoms were observed jumping on top of the gold flake, after which they can rejoin the flake at an edge.

The stability of the monolayer Au flakes was further tested under continuous electron beam irradiation. The rotation of the cluster and movement of individual Au atoms are clearly visible, as well as a more surprising observation: reversible transformation between the flat gold structure and a three-dimensional (3D) amorphous cluster. This is in contrast to the 2D CuAu structures, which were observed to collapse and turn into a 3D cluster, but never the other way around.^19^ An example image sequence is shown in Figure 2d and in video V2 of the Supporting Information. Here, an initially 2D structure turns into a quasi-spherical nanocluster during imaging. Then, after remaining in the 3D shape for ca. 51 s, it unfolds again into a monolayer (the frame time is ca. 4 s). An initially 3D nanocluster was never observed to unfold during imaging. The transformation in both directions is likely triggered by the electron beam that can deliver elastically a maximum of ca. 0.71 eV to a gold atom at 60 kV.

### Multilayer Gold Platelets

Upon increasing the irradiation fluence, the Au clusters grow in thickness and in size up to several tens of nm in diameter. They have a distinct flat structure and occur in different crystal orientations. An overview of an area with several nanoflakes is shown in Figure 3a and an example of a flake with a honeycomb structure with a lateral extent of approximately 10 nm is shown in Figure 3b. Moreover, flakes with a hexagonal arrangement of Au atoms can also be formed, as shown in Figure 3c. The simulated STEM images for two- and three-layered structures viewed from the (111) direction of an FCC lattice presented in Figure 3d reveal structures in Figure 3b,c to likely be a double layer and triple layer, respectively. Regarding the honeycomb structure, a lattice parameter of 0.284 ± 0.021 nm is measured (Figure 3e). In contrast to the atomically thin gold, these thicker flakes remain stable under the electron beam at both 40 and 60 keV. Only at the edges the structure appear to be less stable, resulting in occasional local rearrangement and even local amorphization during continuous imaging.

**Figure 3:**
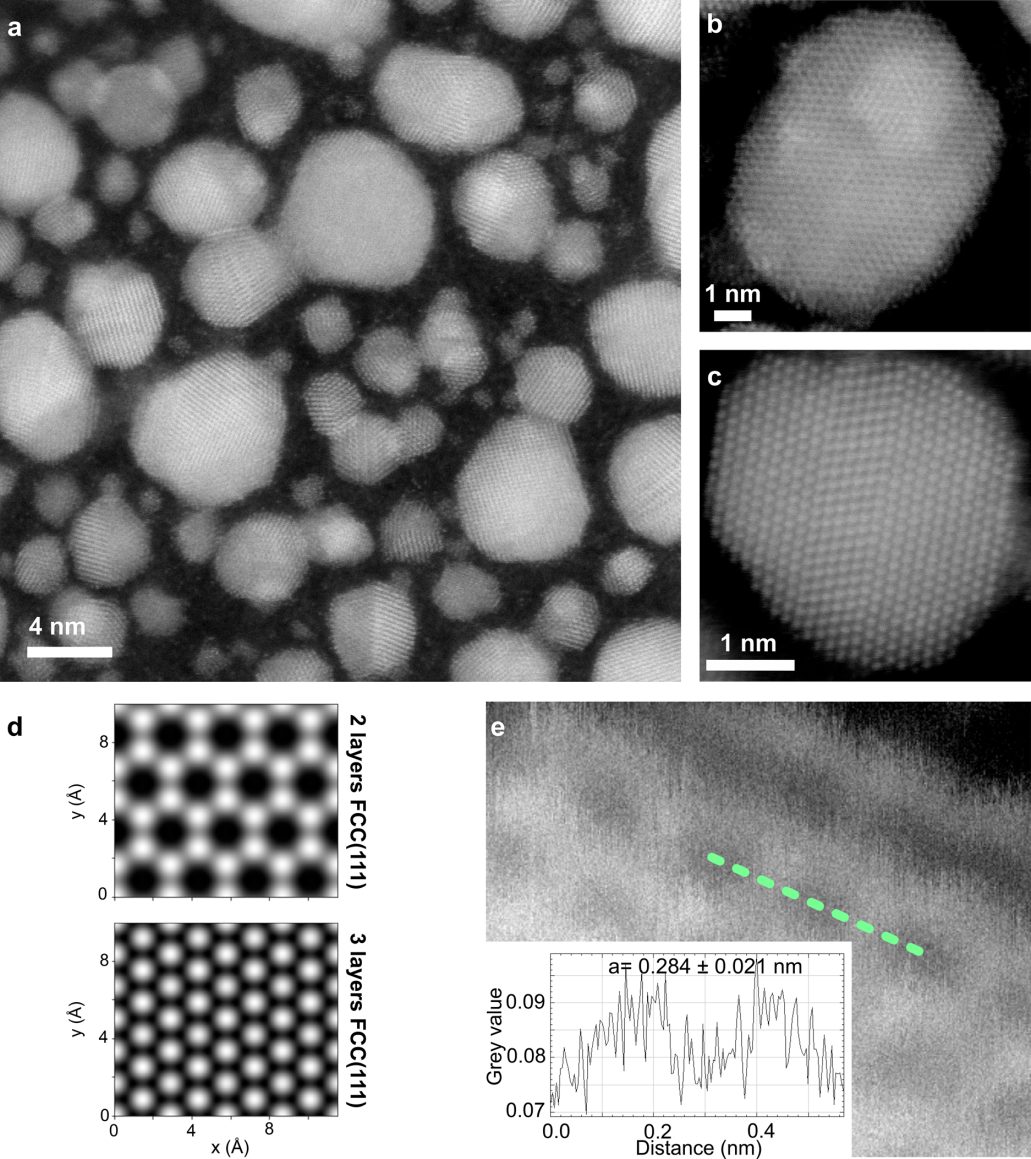
Multilayer flat gold platelets. (a) Overview of the sample with several nm-sized clusters grown during implantation with a high Au dose (1 × 10^16^ cm^–2^). (b) MAADF STEM image of flat multilayer gold structures with a honeycomb lattice, likely a double layer, and (c) hexagonal lattice with two twin boundaries and three or more atomic layers thick. (d) MAADF STEM image simulations of the FCC(111) orientation with two- and three-Au-monolayer-thick structures. (e) Magnification of the honeycomb lattice. An example intensity line profile is shown in the inset. Based on many similar measurements, the lattice parameter is 0.284 ± 0.021 nm.

In order to observe the dynamic behavior in detail, a flake with a few atomic layers (Figure 4) was chosen and imaged continuously with a 40 keV electron beam. The left edge of the flake is attached to a larger gold platelet visible as the bright part of the images. Such proximity to a large cluster increased the stability of the flake, allowing its continuous imaging. As in practically all images shown here, due to the bright scattering contrast of gold, the graphene underneath appears black when the contrast is adjusted to make the gold structure visible. At the edge of the flake highlighted with a green dotted line, the structure is likely to contain two atomic layers. In this area, atom-by-atom reduction and growth is observed. The gold features either disappear completely or lose brightness at the edge (leaving a single Au), as seen in frames 15 and 45. The edge is at its largest in frame 45 and has shrunk back to the original size in the last shown frame (no. 73). In the area highlighted by the orange dotted line, rearrangement of the lattice is observed. Based on the contrast, this part of the structure is several atomic layers thick. In frame 12, the bright atoms display almost a square arrangement, possibly due to a tilt of the structure with respect to the electron beam. In frames 15 and 45, the contrast of the atoms is slightly weaker, and the highlighted area is less organized. By frame 73, the area has rearranged into the honeycomb symmetry typical for double-layer FCC(111). The whole dynamics can be found in video V3 of the Supporting Information.

**Figure 4:**
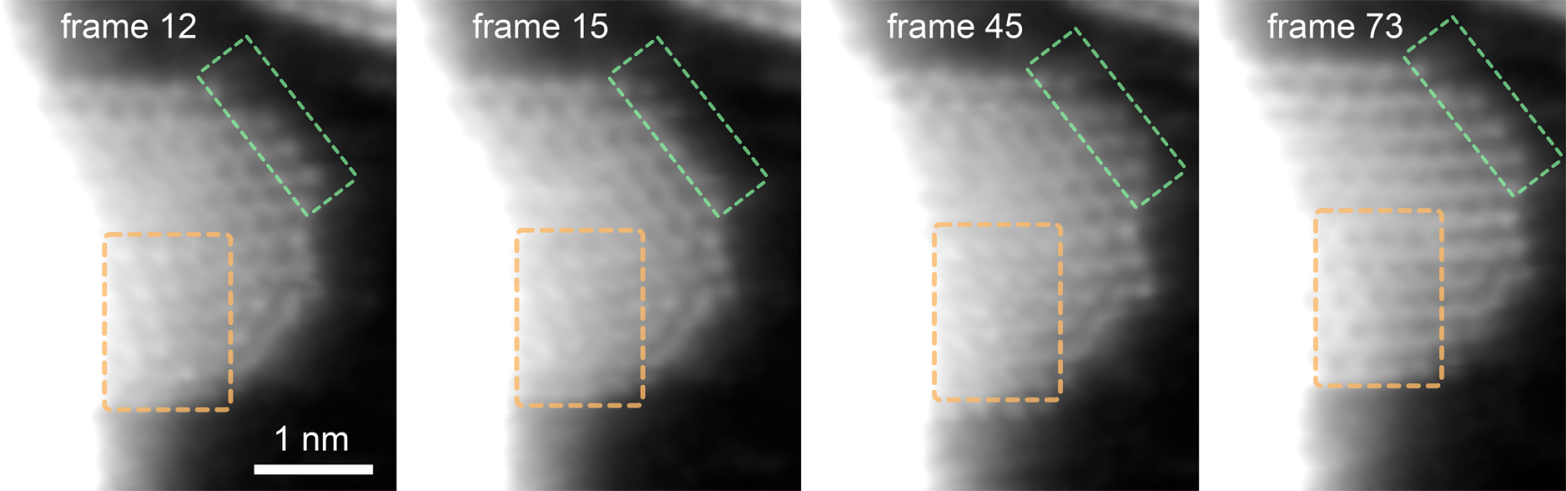
Electron beam-driven dynamics of a few-layer Au platelet. Selected frames highlighting the dynamic transformation in two areas of the gold flake acquired at 40 keV electron beam energy. The area encircled with green shows atomic growth at the edge. The area encircled with orange shows atomic rearrangement. The overexposed area on the left denotes a thicker gold platelet partially on top of the flake.

### Stability and Dynamic Behavior of 2D Gold

The binding energy for varying sizes of one-atom-thick 2D gold clusters formed on a pristine graphene substrate and a graphene substrate with a vacancy has been first calculated using density functional theory (DFT) within a generalized gradient approximation (GGA) for the exchange correlation function and is shown in Figure 5a. A single gold atom binds to a vacancy with ca. −3 eV, compared to less than −0.5 eV for the same atom on pristine graphene (Figure 5a). When the first atom is anchored to the vacancy, adding more atoms lowers the binding energy per atom, but it remains lower by ca. 0.5 eV than the binding energy of a structure with the same size on pristine graphene for at least up to seven atoms.

**Figure 5:**
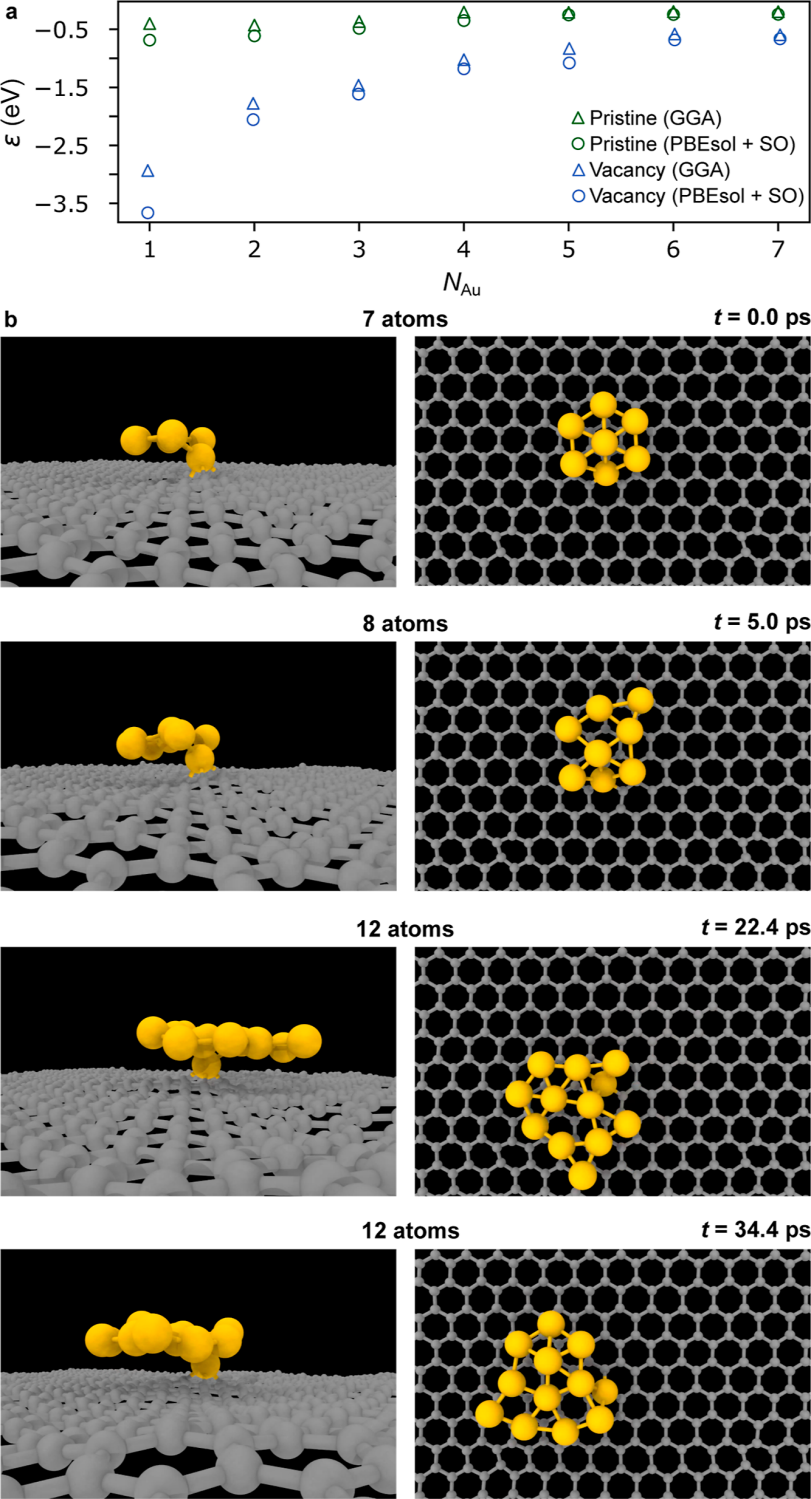
Ab initio calculations of 2D gold. (a) binding energy per atom ε as a function of the number of gold atoms *N*_Au_. (b) Snapshots acquired from dynamic simulations of 2D gold growth with gold atoms thermally migrating across the graphene surface to form a flat 2D structure. The side view is presented on the left and the corresponding top view on the right.

This explains why defect engineering is crucial for the growth of goldene on graphene, a process that is extremely unlikely for gold atoms migrating on pristine graphene (neglecting other irregularities such as contamination, folds or grain boundaries) due to the lack of trapping sites. On defective graphene, metal atoms are trapped by vacancies and can serve as seeds for the growth. The calculations were repeated with the Perdew–Burke–Ernzerhof exchange correlation function for solids (PBEsol), including noncollinear spin–orbit coupling (SO), which includes relativistic considerations and has been shown to be a reliable approach.^35^ Despite a slight offset in the absolute values, the trend with increasing number of gold atoms is similar (also shown in Figure 5a). The stability of even larger 2D flakes was tested with both GGA (48 atoms) and PBEsol (13 atoms) approaches. Both methods indicate the stability of the 2D structure at room temperature and are in agreement with earlier computational data.^15,36^

The bonding between the 2D gold flake and the graphene was investigated by looking at the charge density. The difference calculated between the charge density of the gold flake anchored in graphene and that of a hypothetical system constructed from a superposition of free atoms reveals the bond type. The analysis shows a considerable accumulation of electron charge along the Au–C bonding directions at the vacancy site, i.e., between the anchored Au atom and the carbon atoms, see Figure S4 in the supplement. This is indicative of a directional bond and, therefore, covalent bonding. Such a pile-up of electron density cannot be observed between the other Au atoms in the flake and the carbon atoms in this defective graphene system nor along Au–C bonds in the gold on pristine graphene. The reported electron exchange of a singleAu atom embedded in a graphene vacancy with the neighboring C is small, 0.134 e^–^ per C (Au in a single C vacancy) and 0.127 e^–^ per C (Au in a double C vacancy).^25^

The snapshots of dynamical simulations showing the growth process of 2D gold are displayed in Figure 5b. It starts with the seven-atom flat structure anchored to a vacancy (*t* = 0.0 ps) for which the value of the binding energy is included in Figure 5a. Next, gold atoms are added at room temperature as adatoms thermally migrating across the graphene surface until they encounter the gold flake, where they will bind to it. The first additional gold atom joins the gold flake after 5.0 ps. This step is repeated multiple times until, at 22.4 ps, the gold flake has grown to a total of 12 atoms. At 34.4 ps, the structure of 12 gold atoms remains flat even after the relaxation time of 12.0 ps. The dynamic simulation is shown in video V4 of the Supporting Information. This simulation corresponds to the case where, during the irradiation, the gold atoms land on graphene far away from the flake (which, statistically speaking, is true for most of the impinging gold atoms), fully dissipating their kinetic energy before thermally migrating on the surface. However, close impacts to the flake cannot be fully ruled out, which is why the dynamic simulations for close impacts with a kinetic energy of 25 eV (see video V5 of the Supporting Information) were also performed. The simulation starts with a graphene lattice with a vacancy. It is filled by the first closely impinging gold atom after 0.4 ps, which then acts as the nucleation site for the following growth. Additional gold atoms are introduced at intervals shorter than 1 ps, which is enough to allow the structure to equilibrate. Contrary to the previous case, during the course of the irradiation, the kinetic energy of the ions now causes the structure to be disrupted, and it occasionally starts to form an amorphous quasi-spherical cluster. Nevertheless, even in these dynamic simulations, the quasi-spherical structure relaxes to a flat configuration in a few picoseconds after the last gold atom impact.

The fact that an amorphous cluster can fall into a flat configuration and remain flat implies that the 2D structure is a (meta)­stable configuration for gold atoms. This simulation also gives an explanation for the experimental observation of the reversible 2D → 3D transformation: In the simulations, it can be triggered by the energy provided by the added gold atoms (25 eV), and during the imaging, by the electron beam.

Further static simulation helps better understanding the energetic differences between 2D gold and 3D gold nanoclusters that have the same number of atoms. In Figure S2 (a 43-atom structure), there is an energy penalty of 2.34 eV when going from the 3D to flat structure on pristine graphene. However, with the presence of a vacancy, the energy penalty reduces to just 0.25 eV. Therefore, it is no surprise that the energy provided by the electron beam can trigger the transformation between these two shapes. Furthermore, despite the somewhat lower energy of the 3D structure, the 3D → 2D transformation may be favored due to the more complicated atomic rearrangement required for turning the flat structure into a 3D shape. Nevertheless, as it was already shown in Figure 2b, both transformations are observed experimentally.

Freestanding goldene may exhibit rippling, but the configuration remains stable and planar even when Au adatoms and impurities are present.^15^ Notably, ab initio calculations indicate^36^ that goldene has high intrinsic conductivity. This opens new avenues for ultrathin electronic devices based on 2D membranes that could utilize very thin membranes of gold synthesized on top.

### Spectroscopic Confirmation of Chemical Composition

EELS was used to confirm the chemical identification of the bright atoms in the flat structures. The presence of Au can be seen from the gold *O*_3_ and *O*_2_ peaks at 54 and 72 eV, respectively. However, graphene and gold plasmon peaks appear at similar energies, complicating the analysis, which necessitates deconvolving the spectrum to remove the background that makes up much of the measured signal, as shown in Figure 6a. These include the graphene π and π + σ plasmons as well as several plasmon peaks that are attributed to 2D gold and other gold structures in the vicinity of the sample location under investigation (plasmonic excitations can be detected at distances on the order of several nm^37^).

**Figure 6:**
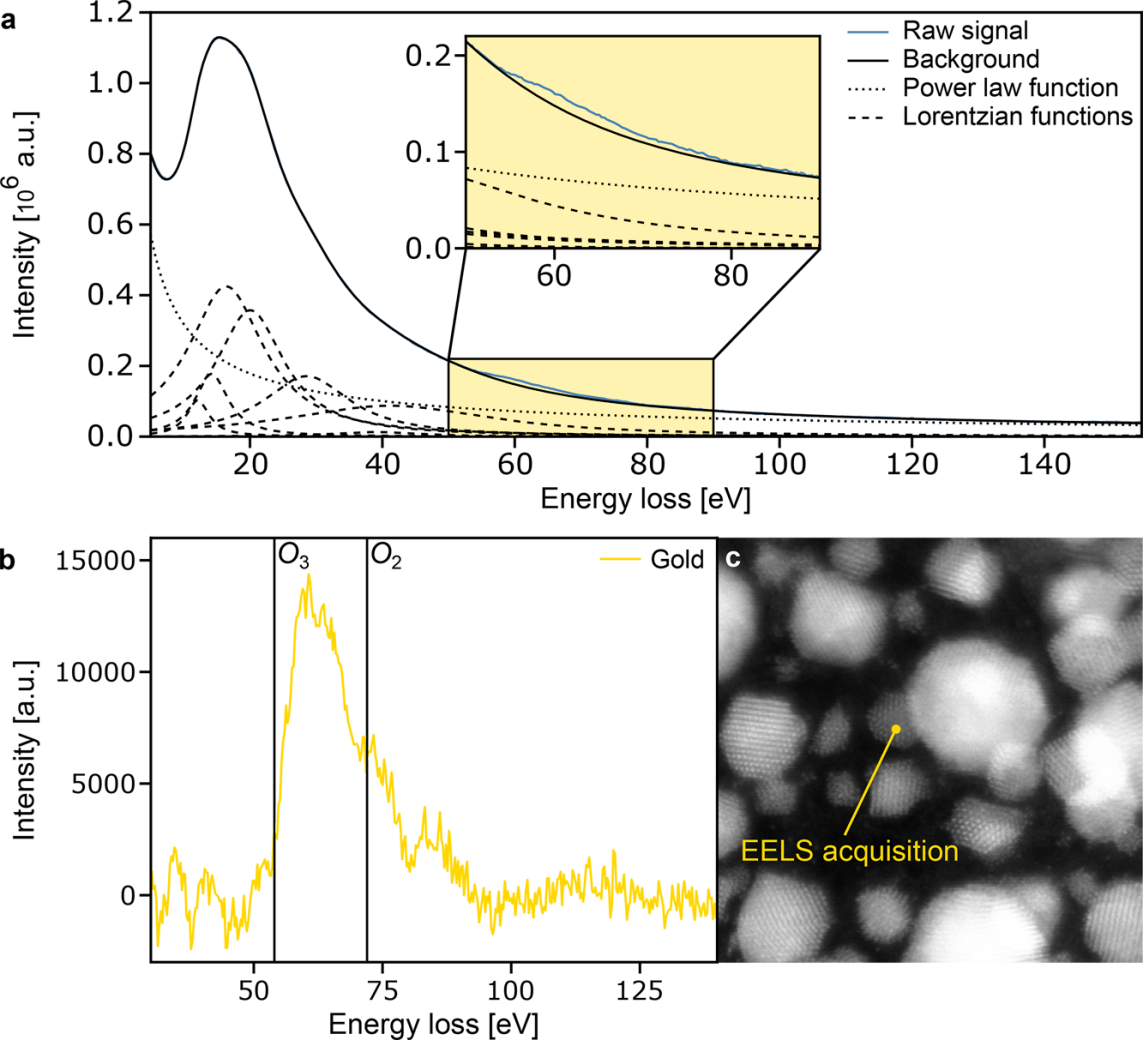
EELS of 2D gold flake. (a) EELS spectrum of a thin gold flake supported by monolayer graphene composed of a power law component, several Lorentzian functions, and the *O*_2_ and *O*_3_ edges of gold. The magnification marked in yellow highlights the gold signal emerging from the background. (b) Gold signal after background subtraction. (c) MAADF STEM image of the acquisition location.

The gold signal after background subtraction shown in Figure 6b contains visible *O*_3_ and *O*_2_ edges. Details about the background subtraction are explained in the methods section and in Figure S3. In short, the background was subtracted by carrying out deconvolution using both a purely mathematical approach and fixed Lorentzian peak positions at known plasmon energies. Overall, in both cases, the resulting deconvoluted spectrum clearly shows the Au *O*_3_ and *O*_2_ peaks, as expected for a gold structure. Figure 6c shows the location on the sample where the EELS spectra were acquired. Fe and Cu can sometimes be found in CVD-grown graphene samples. Therefore, it was confirmed that they are not present in the gold flakes (neither iron *L*_2_ at 721 eV or *L*_3_ at 708 eV nor copper *L*_2_ at 951 eV or *L*_3_ at 931 eV were found). While this means that the bright atoms in the observed flakes indeed are Au and not Fe or Cu, this does not exclude their role as the occasionally observed impurity atoms (which were tentatively identified as Cu).

## Conclusions

2D monolayer gold structures were synthesized on freestanding defect-engineered graphene in the form of one-atom-thick flakes that have a diameter of several nanometers. Defect engineering and Au deposition were carried out with low- (200 eV) and ultralow- (25 eV) energy ion irradiation. Simulations were carried out to confirm the necessary role of carbon vacancies in the growth process. Specifically, they showed that while a 3D cluster is energetically significantly more stable on a pristine surface, this difference diminishes when a defect is introduced in graphene. Due to the similar energies of 2D gold and 3D gold nanoclusters of the same size, they can undergo reversible transformations between the two shapes due to electron irradiation during imaging. By increasing the amount of gold, the size and thickness of the flakes increase. Few-atomic-layers-thick structures display a dynamic lattice arrangement under continuous imaging, while thicker structures remain stable. Goldene on graphene presents a playground for complex plasmonic interactions that stem from the strong localized surface plasmon resonance of gold in the visible and near-infrared regions that can be harnessed in nanophotonic devices and biomedical systems. Moreover, this synthesis of goldene on graphene demonstrates the possibility for an in-depth understanding of metal contacts grown directly on the otherwise inert surface of 2D materials for nanoelectronics. The goldene/graphene interface itself may provide a highly efficient charge transfer channel for photogenerated carriers for solar harvesting, and the exposed undercoordinated gold atoms provide a highly active system for nanocatalysis, offering interesting possibilities for molecular dissociation for, e.g., the hydrogen reduction reaction.

## Methods

### Sample Preparation

The samples are prepared by transferring CVD-grown monolayer graphene onto a SiN TEM grid or a gold TEM quantifoil grid. For that, commercially available Easy Transfer Monolayer Graphene on Polymer Film by Graphenea, Inc., was used. The graphene was transferred from the polymer film onto the TEM grid via a liquid transfer method. Here, the polymer film was separated from the graphene stabilized by a poly­(methyl methacrylate) (PMMA) layer by submerging it in deionized water, which results in the graphene/PMMA structure floating on the surface. The floating structure is then scooped out onto the TEM chip. Following the transfer onto the grid, the graphene is annealed on a heating plate at 150 °C. After annealing, the PMMA layer was dissolved in an acetone bath at 50 °C over the course of 1 h. The sample was then immersed in an isopropyl alcohol bath at room temperature for 1 h.

The SiN grids were manufactured by Silson Ltd., consisting of a 3 × 3 array of SiN support windows on a Si wafer frame. Each of the 9 support windows with 1 μm thickness has up to 25 circular perforations with a diameter of 3 μm. The gold TEM grids are 200 mesh grids with an amorphous carbon quantifoil layer on top containing micrometer-sized holes. The graphene transferred on top of the grids was therefore locally freestanding.

### Ion Implantation

The gold was implanted with the KIIA 500 kV ion implanter at the Helsinki Accelerator Laboratory. The samples were first heated on a hot plate in air for 1 h and then inserted in the sample chamber for implantation. The vacuum was pumped down overnight to reach 10^–7^ mbar. The ion implanter produces a beam of accelerated and mass-selected ions that are then decelerated to the selected energy just before they arrive at the sample one at a time.

The implantations were done by applying two consecutive implantation steps without removing the sample from the vacuum in between. In the sample with the lowest dose, resulting in the one-atom-thick gold, the defects were created using 200 eV ^197^Au^+^ with the dose of 1 × 10^14^ cm^–2^, and the gold was implanted using 25 eV ^197^Au^+^ with the dose of 1 × 10^14^ cm^–2^. The second lowest dose resulting in multilayer gold was achieved using 200 eV ^197^Au^+^ with the dose of 1 × 10^16^ cm^–2^, and the implantation of gold was done with 25 eV ^197^Au^+^ with the dose of 1 × 10^16^ cm^–2^. Two more samples with higher doses were created by repeating this cycle two and three times, respectively. These doses caused severe damage to the freestanding graphene, resulting in a partial breakdown of its structural integrity, but nevertheless, parts of the freestanding graphene membrane at the rim of the holes remained intact, hosting the goldene flakes. The beam current was approximately 3 × 10^–8^ A, measured in a “shoot through” doughnut-shaped Faraday cup placed before the sample. The samples were transferred to the microscope under ambient conditions for the analysis.

### STEM ADF Imaging

The atomic resolution images were acquired via STEM performed on a Nion UltraSTEM 100 by Bruker Corporation operating at 10^–10^ mbar. Upon loading the samples in the vacuum system, they underwent annealing at 160 °C for 10 h to reduce hydrocarbon contamination. The atomic structures were imaged using acceleration voltages of 40 and 60 kV in an annular dark-field imaging mode. The scattered electrons are detected via a MAADF detector and a HAADF detector with a semiangle range of 60–200 mrad and 80 −300 mrad, respectively.

### EELS

The EELS spectra were recorded with a Gatan PEELS 666 spectrometer with an Andor iXon 897 electron-multiplying charge-coupled device camera. The energy dispersion was 0.3 eV/pixel. Multiple point spectra as well as spectral maps of 32 × 32 pixels were collected at various locations on the sample.

The presence of Au is related to the *O*_3_ and *O*_2_ edges, which are observed in the spectrum at 54 and 72 eV, respectively, as shown in Figure 6b, confirming that the platelets consist of gold. At low energies, gold has distinct plasmons that overlap with the edges and add a layer of difficulty in extracting the signal. To mitigate this, deconvolution of the background signal was performed using the eXSpy functionality of the HyperSpy package. The deconvolution considers a total of eight components: a power law and 7 Lorentzian functions accounting for plasmonic activity at and near the point of acquisition. The latter is defined as

1 $$I\left(e\right)=\frac{A}{\pi }\frac{\gamma }{{\left(E-E_{0}\right)}^{2}+\gamma^{2}}$$

where *I* is the spectral intensity and *E*_0_ is the peak position. The parameters *A* and γ are proportional to the amplitude and width of the curve, respectively. Moreover, the power law function used for the deconvolution is defined as

2 $$I\left(e\right)=P{\left(E-E^{\prime }\right)}^{-p}$$

where *P* is the height parameter, *E*′ is the horizontal offset, and *p* is the power law exponent. The parameters of interest obtained by eXSpy are imported to a Python script, and minor adjustments were performed resulting in the background fit displayed in Figure 6a.

Regarding the power law component, the parameters of *P* = 2.08 × 10^6^, *E*′ = 0.2 eV, and *p* = 0.82 were used. The parameters of the Lorentzian components are displayed in Table 1. Due to some inconsistency in the scaling, the raw signal had to be retroactively multiplied by a factor of 3.39 to match the sum of all components, as shown in Figure 6a. While the provided parameters return a mathematically satisfactory fit to the background signal, they cannot confidently assign Lorentzian components to real individual plasmons. The difficulty in an accurate determination of the plasmonic activity comes from the large number of gold nanoparticles in the vicinity of the spectrum acquisition shown in the MAADF image in Figure 6c. The delocalization of plasmonic excitations over lateral extents in the order of nm^37^ opens up the possibility of contributions from the surrounding nanoparticles of different shapes and sizes, resulting in a complex background signal.

**Table 1:** Plasmon Excitations Parameters of the Lorentzian Functions that Represent the Plasmonic Activity of Figure 6a.

	*A*	γ	*E*_0_ [eV]
*L*_1_	6.76 × 10^5^	2.09	12.13
*L*_2_	1.69 × 10^6^	3.03	14.21
*L*_3_	9.26 × 10^6^	6.94	16.40
*L*_4_	7.00 × 10^6^	6.23	20.04
*L*_5_	4.30 × 10^6^	8.00	28.58
*L*_6_	3.27 × 10^5^	8.00	39.00
*L*_7_	5.22 × 10^6^	19.00	41.15

In order to obtain a better understanding of the background signal, the same deconvolution is performed with fixed Lorentzian peak positions at known plasmon energies. These include the π and π + σ plasmons of graphene and four plasmon peaks that are attributed to the 2D gold flake. The latter were obtained from visual inspection of the spectrum shown in Figure S3a, which was obtained by subtracting an EELS spectrum acquired on the isolated graphene support from the spectrum of interest. Therefore, an attempt for background subtraction was performed considering the aforementioned 6 plasmons with their peak positions being located at 5.3 eV (π), 16 eV (π + σ),^25,38^ 13.4 eV, 21 eV, 29, and 40 eV, resulting in the background fit presented in Figure S3b. This results in suboptimal background subtraction, causing high-amplitude oscillations in the low-loss region, where most of the plasmon activity is present. Comparing the results of both approaches (see Figure S3c) shows that the six plasmons are not sufficient to model the background likely due to unknown contributions from nearby gold flakes of different shapes and sizes leading to an increased complexity. However, the resulting *O*_2,3_ edge structure is identical in both approaches, suggesting that the use of free parameters in the mathematical background subtraction had no impact on the physical features originating from the core loss signal of gold.

### Image Simulations

The STEM image simulations were run with the abTEM package.^39^ The multislice potential had a sampling of 0.02 in the *xy*-plane, a slice thickness of 1 Å (*z*-direction), a finite projection, and the Lobato–Van Dyck parametrization.^40^ The parameters of the probe were selected to match the imaging conditions (energy 40 keV and 60 keV, semiangle cutoff 25, rolloff 0.1, and Cs 1 × 10^5^) while keeping the defocus at zero. Three z-thicknesses (2, 3, and 15 layers) of the simulation cells were selected to represent structures that were close to the ones observed experimentally. The electrons were propagated in this direction.

### Ab Initio Molecular Dynamics

Ab initio molecular dynamics calculations were performed using the CP2K^41^ package based on the PBE functional^42^ and a hybrid Gaussian/Plane-Wave scheme (GPW).^43^ The valence electrons were expanded in double-Gaussian basis sets with one polarization function (DZVP) optimized for multigrid integration,^44^ while the core electrons and nuclei were described by Goedecker-Teter-Hutter (GTH) pseudopotentials.^45^ Four multigrids and a plane wave cutoff of 300 Ry were used in this study. Van der Waals corrections were accounted for using the DFT-D3 method of Grimme.^46^ The calculations were carried out using the *NVT* (canonical) ensemble at 300 K, and the MD time step was set to 0.2 fs. The simulation cell contained 160 carbon atoms in the pristine graphene system.

### Density Functional Theory

Spin-polarized DFT calculations of the binding energies of gold atoms to graphene were performed with the Vienna Ab initio Simulation Package (VASP)^47,48^ within the plane-wave projector augmented-wave method. The structures were relaxed using the Perdew–Burke–Ernzerhof (PBE) exchange–correlation functional^42^ with a force tolerance of 0.005 eVÅ^–1^ and an electronic convergence criteria of 10^–6^ eV. The energy cutoff was set to 550 eV, and a gamma-point-centered Monkhorst–Pack *k*-point grid of 5 × 5 × 1 was used to sample the Brillouin zone. Van der Waals interactions were taken into account using the DFT-D3 method^46^ with the Becke–Jonson damping function. The pristine graphene supercell contained 98 and 392 carbon atoms in the binding energy calculations of Au_*n*_, *n* ∈ [1, 7], and structural relaxation of Au_43_ clusters/flakes, respectively.

The binding energies were calculated as follows

3 $$E_{binding}\left(n\right)=\frac{1}{n}\left(E_{graphene+Au_{n}}-E_{graphene}-E_{Au_{n}}\right)$$

where *n* is the number of gold atoms in the cluster, $E_{graphene+Au_{n}}$ is the total energy of the gold cluster on the graphene sheet, *E*_graphene_ is the total energy of only the graphene sheet, and $E_{Au_{n}}$ is the total energy of the isolated gold cluster in the gas phase.

## Supplementary Material

















## Notes

MAADF STEM image series showing minor translation of the 2D gold flake across the graphene surface suggesting weak bonding to the substrate (AVI)
Full MAADF STEM image series showing a reversible transformation from a flat gold structure to a spherical amorphous structure caused by the electron beam for Figure 2d (AVI)
Full MAADF STEM image series of electron beam-driven dynamics of a few-layer Au platelet for Figure 4 AVI file 3.(AVI)
Dynamic simulations (side view) of the growth of a 2D gold flake enabled by gold atoms thermally migrating across the graphene surface (MP4)
Dynamic simulations (top view) of the growth of a 2D gold flake enabled by gold atoms thermally migrating across the graphene surface (MP4)
Dynamic simulations (side view) investigating the effect of nearby impacts of gold atoms with kinetic energies of 25 eV (MP4)
Dynamic simulations (top view) investigating the effect of nearby impacts of gold atoms with kinetic energies of 25 eV (MP4)
Snapshots at different times acquired from V5 and V6 showing the reversible disruption of the flat structure into a spherical structure, simulation results of the influence of a vacancy on the energetic differences between a flat and a spherical gold structure, further details on the background subtraction approach applied for the atomic resolution EELS, and visualization of the charge density calculations
(PDF)
