# Supplementary material for: Two-Dimensional One-Atom-Thick Gold Grown on Defect-Engineered Graphene
Source: ACS Nano. 2025 Jun 10;19(24):22032–43. doi: 10.1021/acsnano.5c01538 (PMC12203644; doi:10.1021/acsnano.5c01538)
Supplement: Supplementary file 8 [file nn5c01538_si_008.pdf]

Supplementary figures for the manuscript "Two-dimensional one-atom-thick gold grown on defect-engineered graphene"

Wael Joudi[2,3] Sadegh Ghaderzadeh[4] Alberto Trentino[2,3]
Kenichiro Mizohata[1] Kimmo Mustonen[2] Elena Besley[4] Jani Kotakoski[2]
E. Harriet Åhlgren[1,2]

[1]University of Helsinki, Department of Physics, Pietari Kalmin katu 2, 00560 Helsinki, Finland
[2]University of Vienna, Faculty of Physics, Boltzmanngasse 5, 1090 Vienna, Austria
[3]University of Vienna, Vienna Doctoral School in Physics, Boltzmanngasse 5, 1090 Vienna, Austria
[4]University of Nottingham, School of Chemistry, NG7 2RD, Nottingham, UK

* jani.kotakoski@univie.ac.at
† harriet.ahlgren@helsinki.fi

May 9, 2025

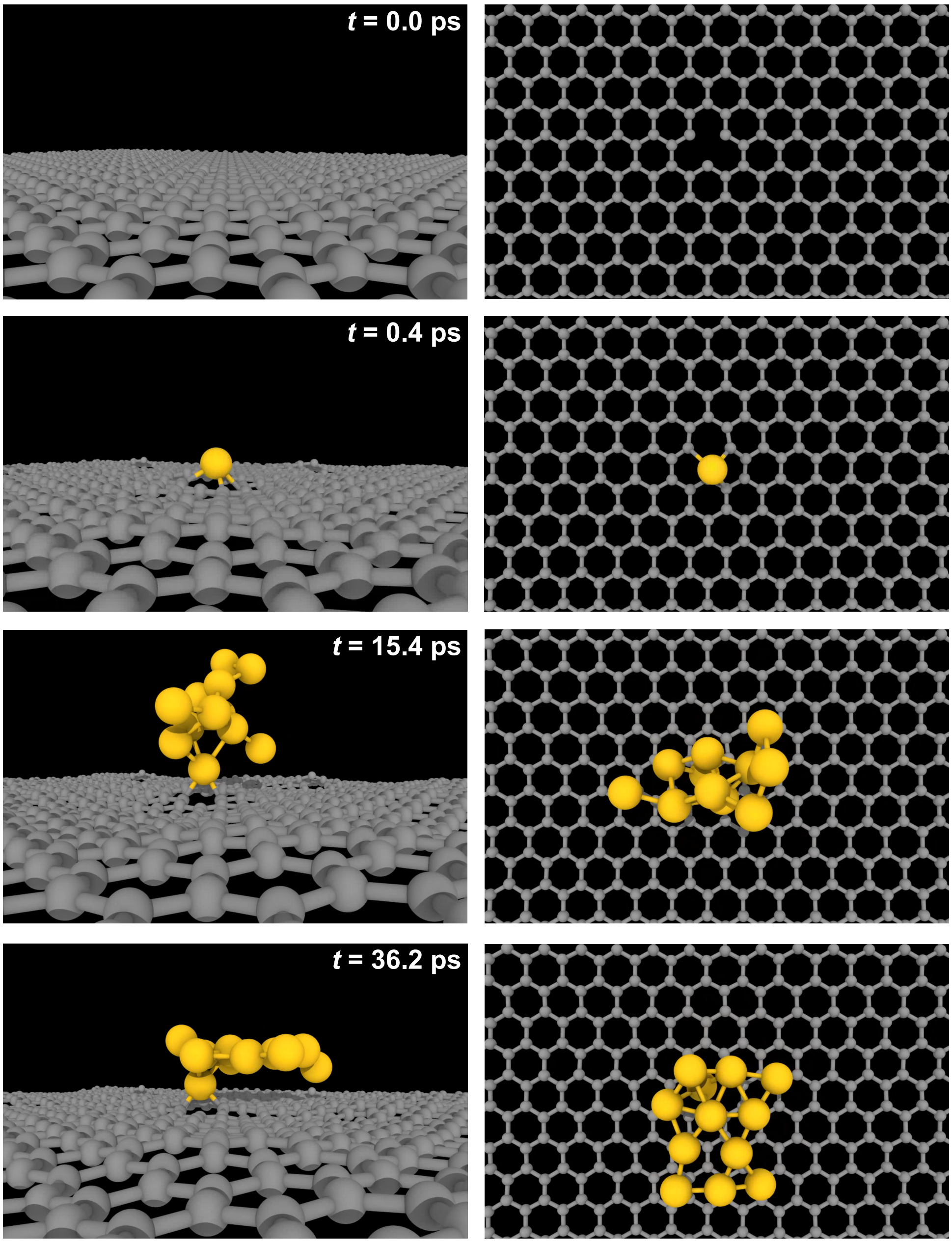

Figure S 1. Dynamic simulation. Snapshots acquired from dynamic simulations mimicking the implantation conditions during the experiment, where the gold atoms impact the graphene with a kinetic energy of 25 eV around the simulation cell.

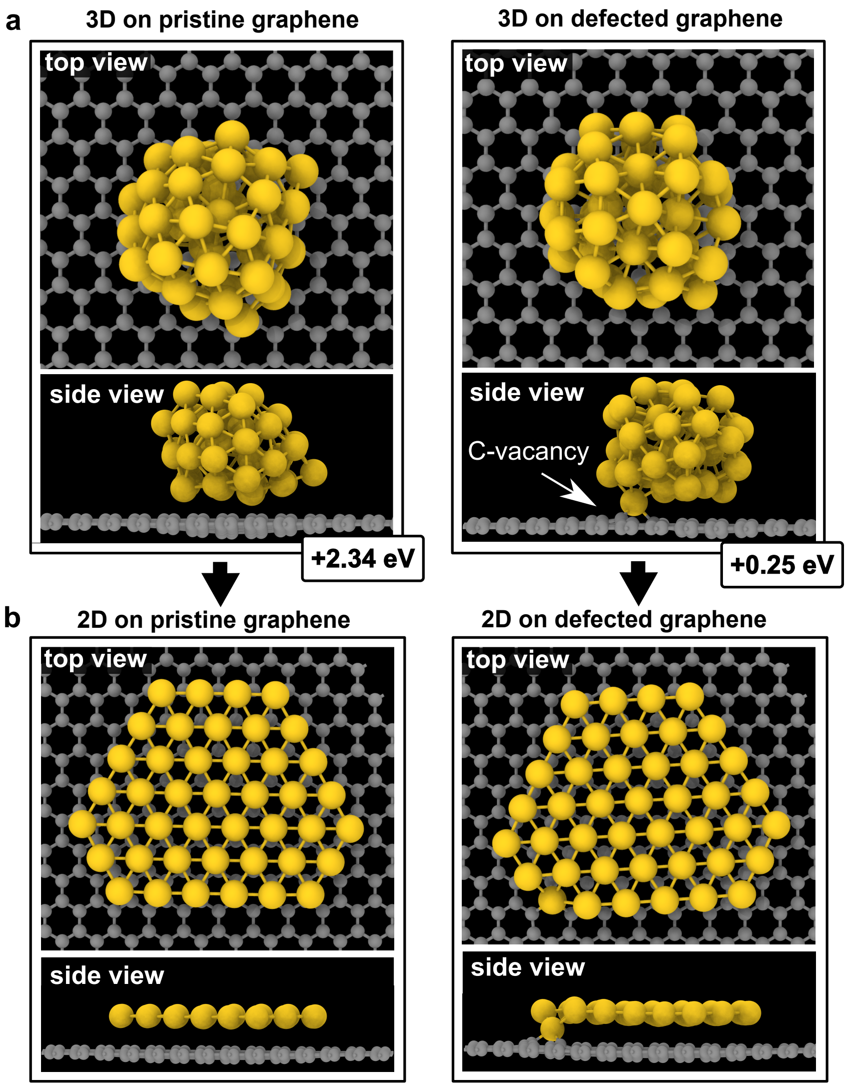

Figure S 2. A spherical gold cluster on pristine (undefected) graphene membrane used as a energy reference. (b) The same cluster in a flat arrangement increases the total energy by +2.34 eV to a less favorable configuration. (c) Optimised structure for graphene with a carbon vacancy supporting a spherical gold cluster. (d) The same cluster in a flat arrangement on defected graphene is only +0.25 eV less favorable than the spherical cluster.

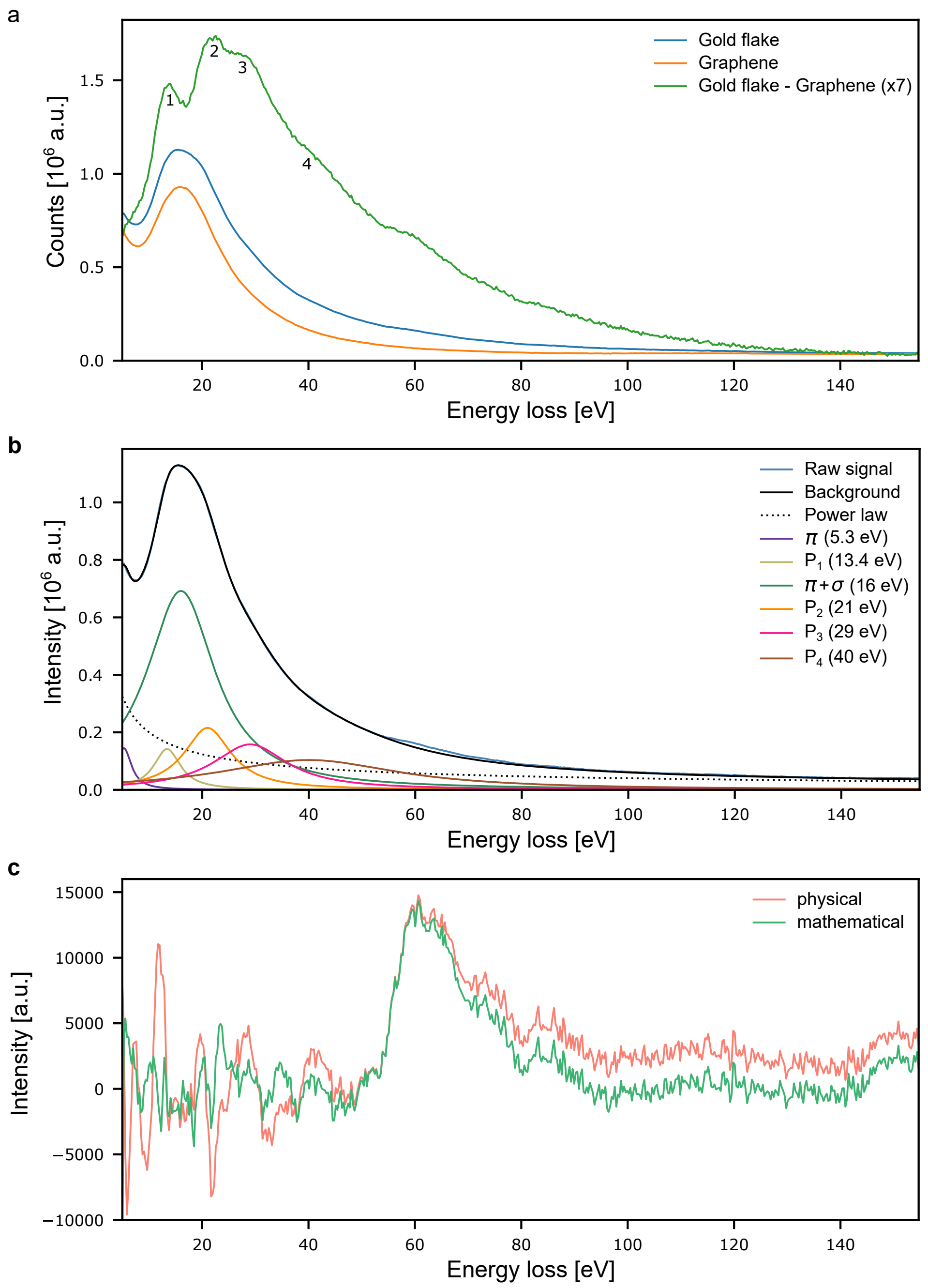

Figure S 3. EELS background subtraction. (a) EELS spectrum acquired on a gold flake supported by graphene (blue), on the graphene support (orange) and the difference of the two (green). The numbers mark the visually observable plasmon peaks originating from the gold flake. (b) Background fit performed including the known graphene π and $\pi + \sigma$ plasmon excitations and the four plasmon peaks determined from a. The energy values in parenthesis represent the peak position. (c) Comparison of the EELS spectra obtained after background subtraction using the physically motivated model presented in b and the mathematical background fit presented in Figure 6a.

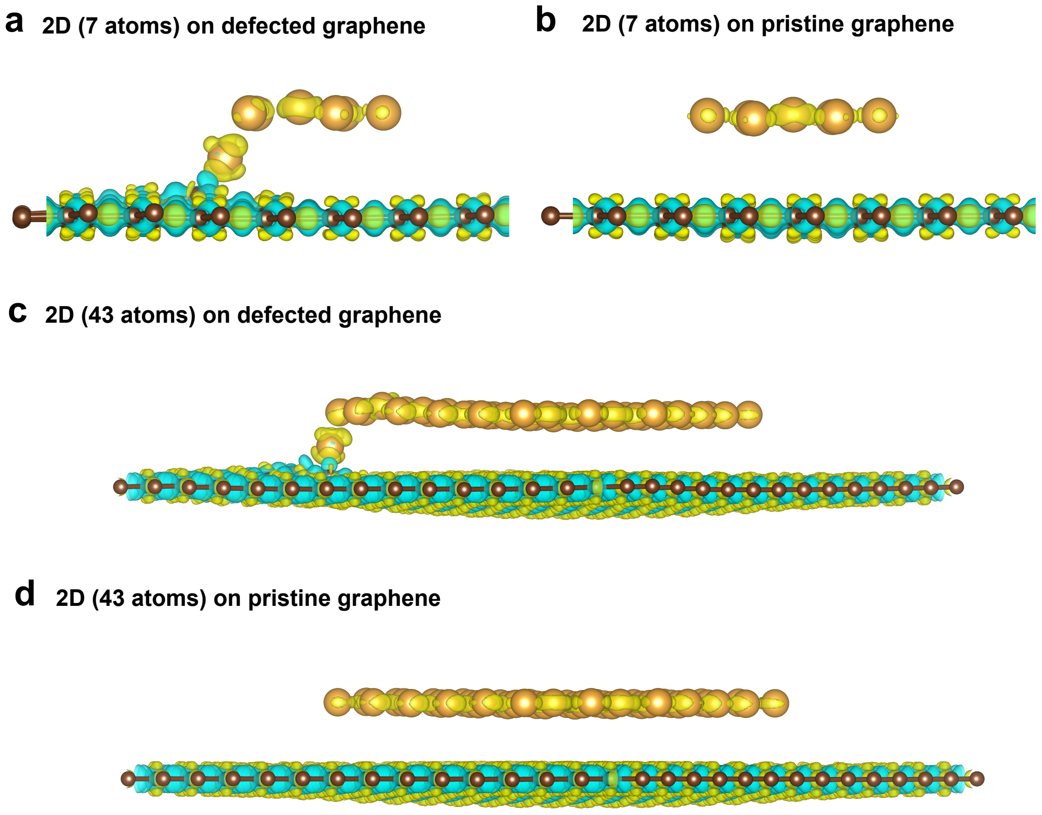

Figure S 4. Charge density. (a) The charge density distribution of a 2D gold flake consisting of 7 atoms on defected graphene indicates covalent boding between the anchoring Au and the neighboring C. (b) Charge density in a 7 atom gold flake on top of pristine graphene. No convalent bondig between any Au and C is detected. (c) Same for larger 43 atom gold flake on defected graphene. Again indication of covalent bonds between anchoring Au and neighboring C is visible (d). 43 atom gold flake on pristine graphene. No indication of increase electron density between Au and C can be seen, hence no covalent bonding between the two can be found.
